# Can we predict the development of acute gastric dilatation in patients with anorexia nervosa?

**DOI:** 10.1186/s40337-023-00937-2

**Published:** 2023-11-29

**Authors:** Kristin Anderson, Ashlie Watters, Elizabeth Dee, Philip S. Mehler

**Affiliations:** 1 ACUTE Center for Eating Disorders at Denver Health, Denver, CO USA; 2 Department of Medicine, University of Colorado School of Medicine, Aurora, CO USA; 3 Department of Radiology, Denver Health Hospital Authority, Denver, CO USA; 4 ACUTE Center for Eating Disorders at Denver Health, 723 Delaware Street, Denver, CO 80204 USA

**Keywords:** Anorexia Nervosa, Acute gastric dilatation, Refeeding, Abdominal pain

## Abstract

**Background:**

Acute gastric dilatation can develop in patients with anorexia nervosa who are being refed to achieve weight restoration. If unrecognized, this condition is associated with significant morbidity and mortality. Patients with acute gastric dilatation usually have abdominal pain, nausea, and vomiting. Abdominal imaging confirms the diagnosis. This study aims to identify risk factors associated with the development of acute gastric dilatation in patients with severe restrictive eating disorders in order to hasten diagnosis and guide treatment. This study also aims to define the clinical outcomes of patients with acute gastric dilatation.

**Methods:**

In this retrospective case series, 15 patients with a restrictive eating disorder were studied. Multiple variables were assessed for significant correlation with stomach size.

**Results:**

15 patients with a restrictive eating disorder were identified as being diagnosed with acute gastric dilatation through chart review during the study period. The average dilated stomach size was 20.5 cm. There was no significant correlation of stomach size with any of the following: % ideal body weight on day of admission, % ideal body weight on day of imaging study, rate of weight gain (kg per week), or duration of illness. Serum levels of sodium, potassium, phosphorus, magnesium, calcium, bicarbonate, blood urea nitrogen, glucose, albumin, and hematocrit on the day of imaging, did not correlate with stomach size. All patients were treated with conservative management. None of the patients required surgical intervention or progressed to gastric necrosis or perforation, and there were no recurrences of the acute gastric dilatation.

**Conclusions:**

There are no specific risk factors significantly associated with the development of acute gastric dilatation in patients with severe restrictive eating disorders. Clinicians should maintain a high index of suspicion for this condition when patients are experiencing abdominal pain, nausea, or vomiting. When promptly diagnosed and treated, outcomes are good. If diagnosis is delayed, the outcome can be dire.

## Abstract (plain language summary)

The mainstay of treatment of patients with severe restrictive eating disorders is initiation of nutrition to gain weight. One potential complication when patients are started on nutrition is the development of a massively enlarged stomach, also called acute gastric dilatation. If a patient’s care provider does not recognize the development of acute gastric dilatation, it can lead to serious problems, including death. Patients with this condition usually have abdominal pain, nausea, and vomiting. These are common symptoms in patients with anorexia nervosa and are often dismissed. Identifying risk factors associated with developing acute gastric dilatation could help providers recognize this condition and promptly start treatment. This study sought to identify risk factors associated with developing acute gastric dilatation in patients with severe restrictive eating disorders. Several variables, including patient age, duration of illness, body mass index, %ideal body weight, laboratory values, medications, type of nutrition, and rate of weight gain were analyzed. This study found that there are no specific risk factors significantly associated with development of acute gastric dilatation in patients with severe restrictive eating disorders being initiated on nutrition. Therefore, providers need to listen to their patients, evaluate symptoms, and have a high index of suspicion for underlying acute gastric dilatation.

## Introduction

Acute gastric dilatation is a potentially life-threatening condition that can arise in patients with anorexia nervosa (AN) who are being refed to achieve weight restoration. In patients with AN, the exact pathogenesis of acute gastric dilatation is unclear, however mechanistic theories include delayed gastric emptying, decreased gastric relaxation, decreased cholecystokinin, and abnormalities in enteric autonomic function [1, 2, 3]. Patients with acute gastric dilatation typically present with the sudden onset of left upper quadrant abdominal pain, nausea, and vomiting, along with abdominal distention. Definitive diagnosis is made by abdominal imaging which shows a massively enlarged stomach. The literature indicates that the mortality rate is around 70% when acute gastric dilatation goes unrecognized and is then complicated by perforation [4, 5]. Most cases of acute gastric dilatation appear to develop in patients with AN binge-purge (BP) subtype, though there are case reports of this condition occurring in patients with AN restricting (AN-R) subtype as well [4]. Despite the few documented cases in the literature, no studies have been done assessing the risk factors associated with the development of acute gastric dilatation in this population. Given the significance of this complication, with associated high morbidity and mortality, this is an area of eating disorders that is deserving of additional inquiry, especially since the vast majority of refeeding occurs in non-hospital settings. This study aims to present a case series about the development of acute gastric dilatation in patients with severe restrictive eating disorders in order to guide treatment and to hasten diagnosis. In addition, this study also aims to define the clinical outcomes of patients with acute gastric dilatation.

## Methods

The ACUTE Center for Eating Disorders and Severe Malnutrition at Denver Health (ACUTE) is a 30-bed inpatient hospital unit that specializes in medical stabilization of patients with severe eating disorders. Admitted patients are generally less than 70% of their ideal body weight (%IBW) and have significant medical complications from their malnutrition. A multidisciplinary team initiates medical stabilization and nutritional rehabilitation. The majority of patients are started on 1400 calories (kcals) by mouth (PO) on admission. Kcals are increased by 300 kcals every 3–4 days until meeting the unit’s expected weight gain trend of 0.2 kg/day. This retrospective case series included 15 patients who admitted to ACUTE between January 2017 and May 2022. Demographic data are listed in Table 1. All included patients were at least 18 years old, diagnosed with AN-R, AN-BP, or avoidant restrictive food intake disorder (ARFID) via the DSM-5 [6], and were found to have acute gastric dilatation on abdominal imaging. Abdominal imaging included a kidney, ureter, and bladder (KUB) X-ray or a computed tomography (CT) scan of the abdomen with or without contrast. Patients either underwent abdominal imaging to further investigate symptoms of abdominal distention, abdominal pain, nausea, and/or vomiting, or for non-symptom-based reasons (i.e. to confirm Dobhoff tube placement for tube feeding) and were incidentally found to have acute gastric dilatation. A radiologist confirmed acute gastric dilatation by measuring the gastric length on KUB and by measuring the gastric dimensions in the anteroposterior (AP), coronal, and sagittal planes on CT scan.

All data, including demographic and anthropometric data, laboratory results, dietary intake, average weight gain (kg/week), and abdominal imaging results were queried from Denver Health’s data warehouse or were obtained from manual chart review. Serum laboratory results, if collected within 48 h of imaging, and body weights from the day of imaging were used for analysis. Type of feeding, such as PO or EN, received at the time of diagnosis was obtained via daily dietician progress notes. BMI was calculated as weight (kg)/height (m^2^) and the %IBW was calculated using the Hamwi Eq.  7 Duration of illness was self-reported and recorded on admission. The study was reviewed and approved by the Colorado Multiple Institutional Review Board.

### Statistical analyses

Continuous variables were described using means (M) and standard deviations (SD) or medians and interquartile ranges (IQR) based on normality. *T*-tests were used to assess the difference in %IBW and BMI in patients with gastric dilatation in the first week of admission versus after the first week of admission. Correlations were used to assess associations between stomach size and anthropometric parameters. *P* values of < 0.05 were considered statistically significant, and all analyses were completed using SAS Enterprise Guide software version 7.1 (SAS Institute, Cary, NC).

**Table 1:** Patient demographics (N = 15)

	Mean (SD)	Range
Age	39.1 (11.7)	24–64
Admit BMI (kg/m^2^)	12.7 (1.1)	10.6–13.8
Admit %IBW	59.4 (6.4)	44.0–66.9
Duration of illness (years)	17.4 (9.4)	8–35
Length of hospitalization (days)	37.5 (14.3)	18–67
Kg/wk gained	1.7 (0.8)	0.36–3.6
Stomach size (cm)	20.5 (4.0)	14–26.8
Day of imaging	10.1 (9.0)	0–32
BMI (kg/m^2^) on day of imaging	12.9 (1.5)	9.0–15.4
%IBW on day of imaging	61.4 (6.8)	44.9–74.8
AN subtype	Frequency (%)	
AN–R	6 (40)	
AN-BP	5 (33)	
ARFID	4 (27)	
Gender		
Female	13 (87)	

*BMI* body mass index, *%IBW* percent of ideal body weight, *wk* week, *AN* anorexia nervosa, *AN-R* anorexia nervosa, restricting, *AN-BP* anorexia nervosa, binge purge, *ARFID* avoidant restrictive feeding intake disorder

## Results

The average age of the cohort was 39.1 years (SD: 11.7), ranging from 24 to 64 years, and 87% were female. The average %IBW and body mass index (BMI) on admission were 59.4% (SD: 6.4) and 12.7 kg/m2 (SD: 1.1), respectively. The cohort was comprised of 40% of patients diagnosed with AN-R, 33% with AN-BP, and 26.6% with ARFID.

Of the cohort, 7 (46.6%) underwent initial imaging with KUB, 7 (46.6%) with CT abdomen with contrast, and 1 (6.67%) with CT abdomen without contrast. Of the 7 patients who underwent initial imaging with KUB, 2 patients underwent further evaluation with CT scan of the abdomen with contrast. The decision to obtain further imaging with a CT scan was at the discretion of the attending physician treating the patient at the time acute gastric dilatation was diagnosed. The median day of the hospital stay during which the imaging procedure was obtained was day 7 (IQR: 4–17; range 0–32.) There was no difference in %IBW on the day of imaging between those who were diagnosed with acute gastric dilatation within the first week of admission versus those that were diagnosed with acute gastric dilatation after the first week of admission *t*(12) = 0.43, *p* = 0.67. The average BMI on the day the imaging study was performed, was slightly, but not significantly higher than the admission BMI, at 12.9 kg/m^2^ *t*(12) = 1.25, *p* = 0.23. 93.3% of patients in the cohort were receiving at least one medication known to have the potential to slow gastrointestinal (GI) motility and 78.5% were on two or more of these medications. The most common medications were Zofran (92.8%), an opioid (47.8%), Hydroxyzine (35.7%), and Diphenhydramine (28.5%).

11 of the 15 patients (73.3%) were on 100% PO nutrition at the time of diagnosis of acute gastric dilatation. 2 patients (13.3%) were receiving 100% enteral nutrition (EN). 1 patient was receiving a combination of PO + EN. 1 patient was receiving total parenteral nutrition (TPN) and trickle tube feeds. On the day of diagnosis of acute gastric dilatation, 3 patients were on day one of a kcal increase, 2 patients were on day two of a kcal increase, and the remainder of the patients had been on their current kcal meal plan for greater than two days.

The average dilated stomach size was 20.5 cm (SD: 4.0; range 14–26.8.) There was no significant correlation of stomach size with any of the following: %IBW on day of admission, % IBW on day of imaging study, diagnosis of acute gastric dilatation within one week of admission versus after the first week of admission, rate of weight gain (kg per week), or duration of illness (all *p*’s > 0.05). Serum laboratory levels of sodium, potassium, phosphorus, magnesium, calcium, bicarbonate, blood urea nitrogen (BUN), glucose, albumin, and hematocrit, on the day of imaging, did not correlate with stomach size (all *p*’s > 0.05).

All patients in the cohort were treated with conservative management. Just over half the cohort (53.3%; n = 8) underwent suction via nasogastric (NG) tube and were given a period of bowel rest. Suction was continued in most patients for about 24 to 72 h. Nutrition was reintroduced through clear liquid diet and then advanced back to a regular diet as tolerated. Half of the patients undergoing suction via NG tube were also started on TPN for nutrition support. At discharge, patients were on a PO diet or a combination of PO + EN diet. No patient required TPN at the time of discharge. None of the patients in the cohort underwent surgical intervention for their acute gastric dilatation.

Four of the fifteen patients (26.6%) did not undergo follow-up abdominal imaging with KUB or CT scan of the abdomen after being diagnosed with acute gastric dilatation. Of the eleven patients who did undergo follow-up abdominal imaging, eight patients (53.3%) had no evidence of gastric dilatation on subsequent imaging, one patient (6.6%) had continued gastric dilatation, one patient (6.6%) had probable gastric dilatation, and in one patient (6.6%) the radiologist was unable to accurately comment on stomach size due to massive ascites. However, all of these patients ultimately were able to continue their weight restoration without ongoing evidence of persistent acute gastric dilatation.

## Discussion

A paper recently published by Gibson et al. (2022) [8] sought to define baseline gastric dimensions in patients with eating disorders. This study found that the average stomach size in patients with eating disorders was significantly larger than a control cohort, leading the authors to conclude that malnutrition, resulting from restrictive eating disorders may be associated with an enlarged stomach. These are interesting findings; however, no study has specifically assessed for risk factors associated with acute gastric dilatation in patients with severe restrictive eating disorders. This is the first study to describe a larger case series of patients with severe restrictive eating disorders who developed acute gastric dilatation during their course of weight restoration and medical stabilization.

Interestingly, stomach size did not significantly correlate with any of the variables evaluated in this study. This finding is important because it illustrates that there is no good predictor to definitively alert a clinician to underlying acute gastric dilatation; rather, it emphasizes the importance of clinical bedside evaluation and imaging. This study suggests that acute gastric dilatation may occur at any time frame during a patient’s treatment course, emphasizing the importance of having a high index of suspicion for this condition not only in the inpatient setting, but also at lower levels of care including residential treatment. It also shows that this condition can occur in patients with purely restrictive eating disorders, not solely in patients that engage in binging and/or purging behaviors.

The differential diagnosis for abdominal pain, distention, nausea, and vomiting is broad. Patients with AN frequently experience these symptoms as disorders of gut-brain interaction (DGBI; formally known as functional GI disease) are very prevalent in patients with anorexia nervosa [9, 10, 11] Given this overlap, it can often be difficult to discern between voluntary and involuntary vomiting. However, a patient with AN reporting any of these symptoms should not be ignored, as these symptoms may be the manifestation of acute gastric dilatation. Ideally, clinicians should evaluate these patients at the bedside and pursue abdominal imaging. One can start with a KUB as it can quickly rule in or rule out acute gastric dilatation, allowing clinicians to promptly initiate treatment when indicated. Management includes bowel rest, gentle hydration, possible NG tube placement with suction, and consideration of general surgery consultation. NG tube placement with suction should be considered in patients with severe or intractable nausea or vomiting, and in those patients with abdominal distention on physical examination. Decompressing the stomach through NG suction can alleviate abdominal distention, nausea, and vomiting, which may help reduce the risk of aspiration. In this study there was no difference in management strategy or outcomes in patients who underwent follow-up abdominal imaging after the diagnosis of acute gastric dilatation was made, compared to those patients who did not undergo follow-up abdominal imaging. If patients are stable and clinically improving, it appears reasonable to abstain from follow-up imaging, continuing with medical management and monitoring. This may be especially helpful in residential treatment centers or outpatient settings in which timely access to imaging services and results may not be available. As shown in this study, patients with severe restrictive eating disorders who are diagnosed with acute gastric dilatation can be managed conservatively with good results. None of the patients in this study required surgical intervention and none progressed to gastric necrosis or perforation. If acute gastric dilatation is overlooked, and progression to gastric necrosis or perforation occurs, the outcome is usually dire with a mortality rate approaching 73% [4, 5].

Limitations exist in this study. First, it is a small cohort. Secondly, not all patients in this study underwent follow-up abdominal imaging after the diagnosis of acute gastric dilatation was established and not all patients had an abdominal CT scan. Though all patients in the study clinically improved, without radiologic follow-up we cannot say definitively that this condition had completely resolved. Thirdly, the study patients all had severe forms of their eating disorders and thus the findings herein may not be applicable to those with a milder form of AN.

## Conclusion

Acute gastric dilatation can occur across the spectrum of eating disorders, including restrictive and binge-purge subtypes of AN and in patients with ARFID. There are no specific risk factors significantly associated with the development of acute gastric dilatation in patients with these severe eating disorders.

Most patients with AN undergoing refeeding will experience abdominal pain, bloating, nausea, and/or vomiting at some time during this process. These symptoms should not automatically be attributed to the refeeding process itself or the underlying psychological component of AN, as they may be the only indication of something medically dangerous going on. Patients reporting any of these symptoms should be evaluated at the bedside and abdominal imaging with KUB should be obtained. Clinicians should maintain a high index of suspicion for underlying acute gastric dilatation in patients with severe restrictive eating disorders who are reporting any of the above-mentioned symptoms. As shown in this study, when promptly diagnosed and treated, patients have good outcomes. If symptoms are ignored and the diagnosis of acute gastric dilatation is delayed, the outcome can be dire.

## Data Availability

The data that support the findings of this study are available on request from the corresponding author.

## Abbreviations

AN: Anorexia nervosa
AN–BP: Anorexia nervosa, binge–purge subtype
AN-R: Anorexia nervosa, restricting subtype
%IBW: Percent of ideal body weight
BMI: Body mass index
PO: By mouth
EN: Enteral nutrition
ARFID: Avoidant restrictive food intake disorder
TPN: Total parenteral nutrition
kcals: Calories
KUB: Kidney, ureter, and bladder X–ray
CT: Computed tomography
NG: Nasogastric
DGBI: Disorders of gut–brain interaction
